# Plasma Prekallikrein: Its Role in Hereditary Angioedema and Health and Disease

**DOI:** 10.3389/fmed.2018.00003

**Published:** 2018-01-25

**Authors:** Alvin H. Schmaier

**Affiliations:** ^1^Hematology and Oncology Division, Department of Medicine, University Hospitals Cleveland Medical Center, Case Western Reserve University, Cleveland, OH, United States

**Keywords:** prekallikrein, plasma kallikrein, hereditary angioedema, high-molecular-weight kininogen, factor XII, fletcher trait, thrombosis, bradykinin

## Abstract

Plasma prekallikrein (PK) has a critical role in acute attacks of hereditary angioedema (HAE). Unlike C1 inhibitor, its levels fall during HAE attacks with resultant cleaved high-molecular-weight kininogen. Cleavage of high-molecular-weight kininogen liberates bradykinin, the major biologic peptide that promotes the edema. How prekallikrein initially becomes activated in acute attacks of HAE is not known. PK itself is negatively associated with cardiovascular disease. High prekallikrein is associated with accelerated vascular disease in diabetes and polymorphisms of prekallikrein that reduce high-molecular-weight kininogen binding are associated with protection from cardiovascular events. Prekallikrein-deficient mice have reduced thrombosis risk and plasma kallikrein (PKa) inhibition is associated with reduced experimental gastroenterocolitis and arthritis in rodents. In sum, prekallikrein and its enzyme PKa are major targets in HAE providing much opportunity to improve the acute and chronic management of HAE. PKa inhibition also may be a target to ameliorate cardiovascular disease, thrombosis risk, and inflammation as in enterocolitis and arthritis.

## Introduction

Modern-day hereditary angioedema (HAE), also known as hereditary angioneurotic edema (HANE), credits Quincke for its discovery (1, 2). Shortly thereafter, Osler is recognized for its description (3). It was some time afterward that the mechanistic understandings of the disorder became appreciated. Pensky et al. had a partial purification of a serum inhibitor of C’1 esterase (C1 inhibitor, C1-INH) (4). Landerman et al. suggested that HANE results from a deficiency of an inhibitor for serum globulin permeability factor and/or plasma kallikrein (PKa) (5). Donaldson and Evans were able to connect the dots and deduce that the serum inhibitor to C1-INH was the biochemical abnormality associated with HANE (6). The discovery that C1-INH is the missing factor in HANE changed the name of the disorder to simply HAE.

## Role of PKa in Hereditary Angioedema (HAE)

Ratnoff and Lepow observed that C1-INH enhanced vascular permeability, suggesting that something related to the complement system was etiologic in the angioedema seen in HAE (7). This work confused the scent of investigation as to the agent that induced edema in HAE. It was almost another decade that attention from Landerman’s original report focused on PKa and its importance in HAE. Gigli et al. showed that as PKa activity decreases there is a reciprocal depletion of C1-INH, suggesting that their presence was dependent upon each other (8). Although a number of serpins inhibit PKa, the relative importance of each based upon inhibitory affinity and plasma concentration was shown by Schapira and Colman with the advent of chromogenic assays using peptide substrates. They showed that C1-INH accounts for 42–48% of PKa inhibition and α_2_macroglobulin accounts for 50% (9). All other serpins only account for about 2% of PKa inhibition. Further studies by these investigators showed that C1-INH accounts for 92% serpin inhibition of activated forms of factor XII (10).

With the above background, those investigators asked what is the role of PKa in acute attacks of HAE? It has been previously recognized that C1-INH levels do not change with acute attacks of HAE, although serum C4 is always decreased. It was observed that in acute attacks of Type 1 HAE patients, residual plasma prekallikrein (PK) (zymogen) fell upon disease onset without change in plasma PK antigen (11). This finding means *in vivo* activation of PK to PKa with immediate inhibition of the enzyme by α_2_macroglobulin in the C1-INH-deficiency state. Furthermore, it was observed that both activity and antigen of plasma high-molecular-weight kininogen (HK) were decreased. These data are additional information that *in vivo* activation had occurred. Importantly, factor XII levels were not significantly reduced (11).

The finding that HK activity and antigen were reduced indicated activation. When immunoblotting started, HK was found to be a cleaved protein (cHK) on reduced SDS-PAGE in plasma from HAE patients (12). In a pregnant patient that we studied serially from the 5th month of her pregnancy, we observed that after an HAE attack, she had persistent cHK on reduced SDS-PAGE with reduced plasma levels of PK and HK activity and antigen (13). This patient had persistent, crampy abdominal pain with localized abdominal wall edema for the rest of her pregnancy and spent the last 6 weeks of the pregnancy hospitalized at bedrest with preterm labor. Her symptoms and signs of HAE attacks only resolved in the postpartum period when the patient had normalization of her plasma PK and HK levels with reduction of cHK and the appearance of intact HK on immunoblot (13).

## Role of Bradykinin (BK) in HAE

The finding that circulating HK is mostly cleaved during an attack of HAE indirectly indicates that BK is liberated. By the early 1980s, there were two hypotheses for the edema-producing agent during attacks of HAE. One was a peptide cleaved from C2, C2 kinin; the other was BK. Kaplan et al. showed that BK was in HAE plasma (14). In these experiments, only BK was able to contract rat uterus. Protein C2, C2 cleaved with plasmin, C1 and C2 cleaved with plasmin, C1, C2, and C4 and aggregated IgG, and C1 and C2 cleaved by trypsin did not contain substances that contain the ability like BK to contract rat uterus. Furthermore, heating HAE plasma to 56^o^C did not eliminate the uterine contracting element in HAE plasma (14). Nussberger and Cicardi showed that BK is elevated in patients with acute attacks of HAE and this elevation is localized to certain parts of the body (15, 16). Lastly, Han and Davis showed that in the C1-INH-deleted mice (*serping1^−/−^)*, there is clear evidence for angioedema by the Evan blue dye extravasation and intense vascular permeability. When they mated the *serping1^−/−^* mice with BK B2 receptor knockout mice (*bdkrb2^−/−^*), the angioedema was ameliorated (17).

## Role of PKa in Health and Disease

### Role in Hemostasis

Although PKa is a major enzyme in acute attacks of HAE, what is its physiologic role? Surprisingly, little is really known. Hathaway et al. discovered PK as a missing protein in four members of a family with a prolonged activated partial thromboplastin time (aPTT) (18). It was called Fletcher trait because it was the surname of the affected family. The occurrence in four siblings—offspring of a consanguineous union—suggested autosomal recessive inheritance (19). This defect is characterized by a prolonged aPTT that shortens to normal if the plasma is exposed for an extended period (60 min) to a negatively charged surface such as kaolin or polyP before addition of calcium ions (19, 20). Wuepper showed that the missing protein in Fletcher trait was plasma PK since the purified protein corrected not only the coagulation defect but also the abnormal surface-activated kinin formation and fibrinolysis seen in Fletcher trait (21). Affected individuals do not have a hemostatic disorder, but all surface-activated blood coagulation assays (e.g., aPTT, activated clotting time) are mildly prolonged.

Since in man, a complete deficiency of PK is not associated with abnormal bleeding, this clinical information alone is sufficient to indicate that PK has no role in hemostasis. As a protein participating in surface-activated blood coagulation assays, PK is a weak one. The fact that prolonged incubation on the bench-top shortens the prolonged aPTT in Fletcher trait suggests that PK and its enzyme PKa are not major participants in surface-activated blood coagulation. PK is not essential for contact activation. Meier et al. showed that PK increases the rate of surface-activated blood coagulation, but is not critical to it (22). In the absence of PK, FXII will auto-activate albeit at a slower rate (22). This point is demonstrated in immunoblot cleavage studies with HK. At 1 min, normal and factor XI-deficient plasma HK is cleaved when incubated with kaolin, but not HK, PK or factor XII-deficient plasmas (23). However, at 1 h, HK also became cleaved in PK-deficient plasma, but, again, not in factor XII-deficient plasmas (23).

### Role in Cardiovascular Disease

Human epidemiological studies suggest that constitutively PK itself has a role in cardiovascular disease. In studies on plasma from the DCCT/EDIC Study Group study, elevated plasma PK was associated with hypertension, nephropathy, and accelerated vascular disease in patients with diabetes (24, 25). There is an exonic N142S polymorphism (rs3733402) in Apple domain 2 of plasma PK that results in reduced HK binding in the intravascular compartment (26). This polymorphism is associated with reduced BK formation, lower plasma l-arginine concentrations, and reduced plasma renin (27–29). PK is a plasma prorenin activator. We observed that the SNP rs3733402 confers decreased risk for hypertension and angiographic coronary artery disease in subjects in the PEACE trial (30).

### Role in Thrombosis

Two groups have shown that complete murine *klkb1* depletion (PK knockout) is associated with reduced risk for induced arterial thrombosis (20, 31). Bird et al. observed that thrombus weight and protein content were reduced in *klkb1^−/−^* mice compared with genetic wild types upon ferric chloride administration to the carotid artery (20). Furthermore, blood flow in the injured carotid arteries was prolonged.

Stavrou et al. also observed that *klkb1^−/−^* mice on the ferric chloride-induced carotid artery thrombosis assay had prolonged times to thrombosis (31). Additional studies confirmed that *klkb1^−/−^* mice have reduced thrombosis risk and ascertained the mechanism(s) for thrombosis delay (31). When *klkb1^−/−^* mice were examined on the Rose Bengal carotid artery thrombosis assay, *klkb1^−/−^* mice have twice the time to thrombosis as wild type (*klkb1^+/+^*), and heterozygotes are in-between (*klkb1^+/−^*). When the *klkb1^−/−^* mice were reconstituted with purified normal human plasma PK such that the plasma concentration is 100% normal (~450 nM), the time to carotid artery thrombosis did not shorten (20). This latter observation turns out to be very important since the delay to the time to thrombosis cannot be fully explained by merely the deficiency of plasma PK. Reconstitution of the plasma PK to normal in *klkb1^−/−^* mice corrects its aPTT and contact-induced thrombin generation time to normal, but does not remedy the thrombosis delay. This result is unlike factor XII supplementation that corrects the thrombosis delay in factor XII-deficient (*f12^−/−^*) mice to normal, suggesting that the mechanism(s) for thrombosis delay in *klkb1^−/−^* mice is different than that of factor XII-deficient mice and may not be reduced contact activation.

Studies next determined if there was reduced thrombosis on two contact activation-induced murine models. These models are (1) collagen-epinephrine-induced pulmonary embolism and (2) long-chain polyphosphate-induced pulmonary embolism (20). Our data show that, unlike *f12^−/−^* mice, *klkb1^−/−^* mice do *not* have a survival advantage and reduced pulmonary thrombosis (i.e., less fibrin and platelet deposition in lungs), even though they show reduced contact activation-induced pulmonary vascular leakage (31). This dichotomy of findings (low contact activation, but no protection from thrombosis) indicated to us that another mechanism(s) for thrombosis inhibition was operative.

**Figure 1:**
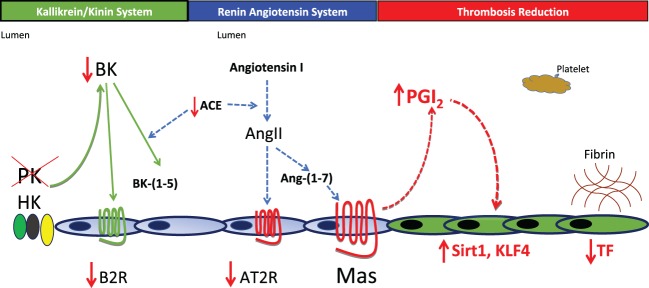
Pathophysiology for thrombosis protection in prekallikrein (PK)-deleted mice. In the absence of PK, there is a decrease in plasma bradykinin (BK) and the BK B2 receptor (B2R). Lower BK and B2R are associated with a reduction in the expression of the renin-angiotensin receptor angiotensin receptor 2 (AT2R). Surprisingly, the Mas receptor becomes overexpressed leading to a 1.5- to 2.0-fold increase in prostacyclin. This elevation of PGI_2_ is not enough to inhibit platelets, but is sufficient to elevate vessel wall Sirt1 and KLF4 that together reduce tissue factor (TF) expression. In this mouse model, reduction of TF appears to be sufficient to reduce thrombosis risk in *klkb1^−/−^* mice.

Another mechanism(s) for thrombosis protection was sought for *klkb1^−/−^* mice. *Klkb1^−/−^* mice have about 50% normal plasma BK levels (31). The kallikrein/kinin system with BK formation and interaction with its receptors does not live in a vacuum (Figure 1). We next observed that the constitutive expression of BK B2 receptor (B2R) was reduced in *klkb1^−/−^* mice. In *klkb1^−/−^* mice, the reduced B2R expression was associated with overexpression of the Mas receptor (both mRNA and protein), with reduced AT2R receptors levels but normal plasma levels of the major ligand of the Mas receptor, angiotensin-(1-7) (31). We observed that in the *klkb1^−/−^* mice, plasma prostacyclin (PGI_2_) also is elevated 1.5- to 2-fold. This degree of elevation of PGI_2_ is not associated with reduced platelet function. In *klkb1^−/−^* mice, CRP-, thrombin-, or ADP-induced platelet activation is normal.

How then did a small elevation of prostacyclin lead to less thrombosis in *klkb1^−/−^* mice? In *klkb1^−/−^* mice, the elevation of PGI_2_ is associated with elevation of two vascular transcription factors: sirtuin (silent mating type information regulation 2 homolog) 1 (Sirt1) and Kruppel-like factor 4 (KLF4). Both regulate NFκB to modulate tissue factor (TF) expression. Sirt1 is a histone deacetylase that degrades p65 in the NFκB assembly and KLF4 binds the p300 coactivator in NFκB. Increased Sirt1 and KLF4 in vessel wall decrease TF mRNA, protein, and activity as measured in a factor X activation assay (Figure 1). This model shows a global pathway for thrombosis risk reduction resulting from absent plasma PK and decreased vessel wall TF (31). Thus, the sum of these investigations indicates that elevated PK is associated with cardiovascular disease, its decrease ameliorates it, and its deficiency is associated with reduced thrombosis risk.

### Role in Sterile Chemical Inflammation Models

Prekallikrein may have an additional role in inflammation. In peptidoglycan-polysaccharide polymer-induced granulomatous enterocolitis, the PKa/kinin system is activated to yield cHK (32). A selective PKa inhibitor (P8720) decreased systemic kallikrein/kinin system activation and ameliorates intestinal inflammation in experimental rat granulomatous colitis (33). More recently, a potent Kunitz-type PKa inhibitor or monoclonal antibody 13G11 to PK suppressed synovial recruitment of endothelial cell progenitors and hyper proliferation of synovial cells in peptidoglycan-polysaccharide polymer-induced experimental arthritis in rodents (34). Lastly, a combined HK (*Kgn1*) and PK (*klkb1*) double-knockout mice have reduced swelling and severity of joint arthritis as well as lower inflammatory cytokines in a rodent model of serum transfer-induced arthritis (35). These animal model studies suggest that PKa is a contributor to sterile, chemical inflammation. These kinds of models best represent mechanisms most applicable to inflammation associated with autoimmune disease.

## Prekallikrein Activation in HAE

Although PKa is important during acute attacks of Type 1 and 2 HAE, the precise inciting etiology for its activation in the disease state is not completely known. Most know that activated forms of factor XII (αFXIIa, βFXIIa) have the ability to activate PK to PKa. The *K_m_* of FXIIa activation of PK and PKa activation of FXII are similar with micromolar affinities. However, acute attacks of HAE are not associated with activation of the blood coagulation system, thrombin formation, and thrombosis. Studies show elevated d-dimer and prothrombin 1 + 2 occurs during the initial phase of an acute attack of HAE, but for the most part these findings are subclinical. Acute attacks of HAE are not associated with thrombosis. The critical question in the contact activation pathway is how does factor XII get activated?

Recent investigations show that there is a single-chain form of factor XII that generates enzymatic activity in the presence of polyphosphates 60–100 molecular units (polyP_60–100_) (36, 37). A single-chain FXII with three arginines in its potential light-chain region mutated to alanines (R353A, R343A, and R353A) incubated in the presence of polyP_60–100_ has the ability to generate amidolytic activity 1/4,000th less than FXIIa. It does require an intact active-site serine (S544). This form of FXII is a single chain on reduced SDS-PAGE and hence has been recommended to be termed scFXII by its creators (36). It has also the ability to activate PK to PKa in the absence of polyP and is able to convert factor XI into factor XIa in the presence of polyP_60–100_ (37). The hypothesis that single-chain factor XII is an activator of PK needs to be demonstrated in plasma *in vivo*. If so, it would suggest that there is continuous potential to form the first molecule of PKa from PK to initiate the kallikrein/kinin system and, in the absence of C1INH, possibly attacks of HAE.

Another hypothesis for PK activation to initiate an acute attack of HAE is that there is an alternative PK activator. This notion is important because up until the last decade, common dogma for the 30 years was that contact activation was only important in mechanical device and infection-related thrombosis. No biologic substance was generally accepted or considered an *in vivo* “contact system” activator. Our laboratory, focusing on the cell biology of the PKa/kinin system and FXII, recognized an endothelial cell serine protease, prolylcarboxypeptidase (PRCP, PCP), that is a plasma PK activator with a *K_m_* of 9 nM (38–40). This enzyme is best known as an endopeptidase with a preferred substrate site of a Pro-X C-terminus. However, our investigations show it is also a stoichiometric activator of plasma PK. In contrast to PK deficiency, PRCP deficiency is associated with hypertension and increased thrombosis risk and reduced vascular cell growth, proliferation, and angiogenesis (41, 42). Presently, it is not known what regulates its endothelial expression and if it is altered in C1INH-deficient patients making them prone to have constitutively increased PK activation. Finally, a second protein, heat shock protein 90 has been proposed as a PK activator (43). Little is known about how it activates PK and what regulates its expression.

## Summary

Plasma PK is a major effector in acute attacks of HAE. How it gets activated to initiate attacks is still not known, but there are several candidate mechanism(s) that need to be examined. Inhibition of PKa in any of the many ways now available should be salutatory to ameliorate disease activity. It is unlikely that single-agent therapy will suppress all attacks under all circumstances. Although we know little about the physiologic role of PKa *in vivo*, its long-term inhibition should be salutatory for HAE prevention, cardiovascular disease risk, and inflammatory disease. Fortunately, we are about to embark upon an era where a plethora of PKa inhibitors will help us to understand how this protein functions in man while improving the care of HAE patients.

## Author Contributions

The author confirms being the sole contributor of this work and approved it for publication.

## Conflict of Interest Statement

The author declares that the research was conducted in the absence of any commercial or financial relationships that could be construed as a potential conflict of interest.

## Abbreviations

HAE, hereditary angioedema; C1 inhibitor, C1-INH, C’1 esterase inhibitor; PK, plasma prekallikrein; PKa, plasma kallikrein; HK, high-molecular-weight kininogen; cHK, cleaved high-molecular-weight kininogen; BK, bradykinin; FXII, factor XII; αFXIIa, βFXIIa, FXIIa, activated forms of FXII; TF, tissue factor; PRCP, prolylcarboxypeptidase.
